# Blueberry Extract and Resistance Training in Severe Pulmonary Arterial Hypertension: Effects on Lung and Right-Ventricle Function and Structure

**DOI:** 10.3390/antiox15101232

**Published:** 2026-09-24

**Authors:** Luciano Bernardes Leite, Leôncio Lopes Soares, Sebastião Felipe Ferreira Costa, Luiz Otávio Guimarães-Ervilha, Sara Caco dos Lúcio Generoso, Mirielly Alexia Miranda Xavier, Arthur Eduardo de Carvalho Quintão, Matheus Soares Faria, Thainá Iasbik-Lima, Flavio Gilberto Herter, Patrick Turck, Alex Sander da Rosa Araujo, Tiago Fernandes, Bruno Rocha de Avila Pelozin, Edilamar Menezes Oliveira, Pedro Forte, Mariana Machado-Neves, Emily Correna Carlo Reis, Antônio José Natali

**Affiliations:** 1 Exercise Biology Laboratory, Department of Physical Education, Federal University of Viçosa, Viçosa 36570-900, MG, Brazilmatheus.s.faria@ufv.br (M.S.F.); anatali@ufv.br (A.J.N.); 2 Department of Physical Education, Federal University of Juiz de Fora, Governador Valadares 36036-900, MG, Brazilmariana.mneves@ufv.br (M.M.-N.); 3 Department of General Biology, Federal University of Viçosa, Viçosa 36570-900, MG, Brazil; luiz.ervilha@ufv.br (L.O.G.-E.);; 4 Faculty of Agronomy, Federal University of Pelotas, Pelotas 96010-610, RS, Brazil; 5 Department of Physiology, Basic Sciences Institute of Health, Federal University of Rio Grande do Sul, Porto Alegre 90010-150, RS, Brazil; 6 School of Physical Education and Sport, University of São Paulo, Av. Professor Mello Moraes, 65-University Village, São Paulo 05508-030, SP, Brazilbruno.pelozin@usp.br (B.R.d.A.P.); edilamar@usp.br (E.M.O.); 7 Department of Sports, Instituto Politécnico de Bragança, 5300-252 Bragança, Portugal; 8 Department of Sports, Higher Institute of Educational Sciences of the Douro, 4560-547 Penafiel, Portugal; 9 Research Center for Active Living and Wellbeing (LiveWell), Instituto Politécnico de Bragança, 5300-252 Bragança, Portugal; 10 Department of Veterinary Medicine, Federal University of Viçosa, Viçosa 36570-900, MG, Brazil

**Keywords:** RV dysfunction, oxidative imbalance, adverse remodeling, myocyte contractility, calcium regulatory proteins

## Abstract

This study evaluated the effects of blueberry extract and resistance training on lung and right ventricle (RV) function and structure in severe experimental pulmonary arterial hypertension (PAH). PAH was induced in male Wistar rats by a single intraperitoneal injection of monocrotaline (60 mg/kg). One day later, the animals started receiving blueberry extract (100 mg/kg daily by gavage) and underwent a three-week moderate-intensity resistance exercise protocol (ladder climbing—15 climbs, a 60 s interval, a 60% load, one session/day, 5 days/wk). After these treatments and echocardiographic assessment, the animals were euthanized, and lung and RV samples were collected for mechanical, morphological, biochemical, and molecular analyses. Both blueberry extract and resistance training attenuated RV dysfunction and pulmonary artery resistance observed over the development of severe PAH. In addition, single RV myocyte mechanical function was maintained in treated rats. Moreover, the applied treatments mitigated oxidative imbalance and adverse structural remodeling in both the lung and RV. Furthermore, the interventions promoted partial or complete preservation of calcium regulatory protein expression. In conclusion, blueberry extract and resistance training, either alone or in combination, alleviate cardiopulmonary oxidative imbalance and adverse structural remodeling, sustain RV myocyte contractility and the expression of calcium regulatory proteins, and thus conserve pulmonary and RV function in severe PAH induced by monocrotaline.

## 1. Introduction

Pulmonary arterial hypertension (PAH) is a rare and progressive disease characterized by sustained increases in pulmonary vascular pressure and pulmonary artery resistance [1,2,3]. This chronic process imposes a progressive overload on the right ventricle (RV), ultimately leading to hypertrophy, dysfunction, failure, and death [1,4,5]. Oxidative stress and inflammation are closely involved in the progression of PAH [6,7,8,9]. In this context, excessive ROS production associated with inadequate antioxidant defenses may promote cellular and tissue injury, contributing to cardiopulmonary dysfunction [10,11,12,13,14]. Within the pulmonary vasculature, oxidative stress in PAH is driven by increased ROS production from multiple sources, including NADPH oxidases, mitochondrial dysfunction, and uncoupled endothelial nitric oxide synthase (eNOS), alongside a reduction in endogenous antioxidant capacity. Elevated ROS levels can decrease nitric oxide availability and favor pulmonary vasoconstriction. At the same time, redox-sensitive signaling pathways may stimulate vascular smooth muscle cell proliferation, inflammatory responses, and extracellular matrix accumulation, thereby promoting pulmonary vascular remodeling [13,14,15,16]. In addition, sustained RV pressure overload may further promote oxidative stress in cardiomyocytes, favoring structural remodeling and oxidative alterations in Ca^2+^-handling proteins that contribute to impaired RV contractile function [10,11,12]. The concomitant inflammatory response can further amplify these alterations, worsening pulmonary vascular and RV dysfunction [15,16]. These complications contribute to the chronic fatigue and impaired quality of life commonly observed in patients with PAH [17].

Despite advancements in conventional treatments for PAH, there are limitations in preventing disease progression, and high mortality and morbidity persist [18,19]. Therefore, non-pharmacological and combined strategies are required to lessen adverse PAH outcomes, like pulmonary artery pressure and RV overload. In this sense, physical exercise [20,21,22,23] and natural antioxidants [24,25,26] have potential as adjuvant components in PAH management. Recent evidence provided by our research group [27,28,29,30,31,32] and elsewhere [9,33,34] indicates that aerobic, resistance, and combined exercise training can preserve cardiac and pulmonary function and structure by counteracting oxidative stress and inflammation, resulting in improved exercise capacity and survival in rats with MCT-induced PAH. Likewise, blueberry extract, which is rich in polyphenols and anthocyanins, can protect cardiac and pulmonary tissues by preventing oxidative imbalance and attenuating functional and structural damage in this model [35,36,37].

Given the therapeutic potential of combining non-pharmacological strategies for severe PAH, previous studies have investigated the effects of exercise training or natural antioxidant interventions separately. However, evidence regarding resistance training and blueberry-derived antioxidants, particularly when assessed alone and in combination through an integrated evaluation of pulmonary and right-ventricular adaptations, remains limited. Therefore, the present study evaluated the effects of blueberry extract and resistance training, alone or combined, on lung and RV structure and function, cardiomyocyte contractility, oxidative stress, and the expression of Ca^2+^-handling proteins in a model of severe MCT-induced PAH.

## 2. Materials and Methods

### 2.1. Experimental Design

Male Wistar rats (~200 g, 6 weeks of age) were randomly distributed into five experimental groups: sedentary control (SC, *n* = 7), sedentary hypertensive (SH, n = 7), sedentary hypertensive blueberry (SHB, n = 7), exercise hypertensive (EH, n = 7), and exercise hypertensive blueberry (EHB, n = 7). Animals were housed in transparent polycarbonate cages (4 per cage) under controlled environmental conditions (23 ± 1 °C, ~60% relative humidity, 12/12 h light/dark cycle), with free access to water and standard rodent chow. All experimental procedures were conducted in accordance with the Ethical Principles in Animal Experimentation and were approved by the Ethics Committee on the Use of Animals of the Federal University of Viçosa (protocol no. 11/2022).

### 2.2. PAH Induction

PAH was induced in the hypertensive groups by a single intraperitoneal administration of monocrotaline (MCT; 60 mg/kg; Sigma-Aldrich, St. Louis, MO, USA), diluted in saline solution (140 mM NaCl, pH 7.4) [38]. Animals assigned to the control group received an equivalent volume of the same saline solution without MCT.

### 2.3. Administration and Phenolic Profile of the Blueberry Extract

Throughout the experimental period, animals in the SHB and EHB groups received a daily oral dose of blueberry extract (100 mg/kg body weight) by gavage at 7:00 a.m. The dose was established based on our previous experimental protocols, in which 100 mg/kg exerted beneficial effects in MCT-induced severe PAH [35,36,39]. In the original protocol, this dose was selected considering the total phenolic content of the extract and the average dietary intake of polyphenols reported for populations in North and South America [39].

The blueberry extract was prepared as previously described [39]. Briefly, fresh blueberries were extracted in 50% ethanol, subjected to mechanical extraction followed by sonication in an ice bath to minimize compound degradation, vacuum filtered, and concentrated using a rotary evaporator. Following preparation, the extract was divided into aliquots and stored protected from light at −80 °C until use. A fresh working solution was prepared daily immediately before oral administration.

The blueberry extract used in the present study was previously characterized in detail [39]. The extract contained 38.89 mg gallic acid equivalents (GAE) of total phenolic compounds per gram. Among the phenolic compounds analyzed, the anthocyanidins cyanidin (219.21 µg/mL) and malvidin (128.68 µg/mL) were detected as the predominant compounds, whereas rutin, gallic acid, catechin, and epicatechin were not detected. Further details regarding the extraction procedure and chromatographic characterization are available in our previous study [39].

### 2.4. Resistance Training Protocol

All animals were adapted to a resistance training protocol (i.e., Ladder climb), as described by Hornberger and Farrar [40]. During a one-week familiarization period, the rats were exposed to the protocol, which consisted of climbing an 80–inclined grid with devices attached to their tails, but without any load. To encourage climbing, a gentle tap was applied to the animals’ tails, and after each climb, they rested for 60 s in a cage (20 cm × 20 cm × 20 cm). This familiarization procedure was performed five days per week. Following the adaptation period, maximum load capacity was assessed before PAH induction and again on days 14 and 21 after induction. The test started with a load corresponding to 75% of body weight, with 30 g increments added on successive climbs. Each attempt was separated by a 120 s recovery interval, and the test continued until the animal was unable to complete the climb.

Resistance training began 24 h after MCT administration and was performed on a vertical ladder adapted for rats. Training loads were individualized according to the maximum load tests, and sessions were conducted five days per week (Monday to Friday) for approximately three weeks. Each session comprised 15 climbs separated by 60 s recovery intervals [41]. Exercise intensity was set at 60% of the maximum load determined during testing, corresponding to a moderate-intensity protocol previously shown by our group to produce beneficial effects in rats with severe PAH [29,31].

### 2.5. Echocardiography

Echocardiographic assessments were conducted on day 23 following MCT administration. Animals were anesthetized with isoflurane (3% induction, 1.5% maintenance; oxygen flow: 1 L/min) and positioned in lateral decubitus. Images were acquired in M-mode (120 frames/s) using a MyLab™30 ultrasound system (Esaote, Genoa, Italy) equipped with a 10 MHz transducers, with the scan speed adjusted to 200 mm/s based on heart rate. Pulmonary artery flow was assessed by pulsed Doppler to calculate the RV acceleration time (AT) and ejection time (ET), with the AT/ET ratio serving as an indirect marker of pulmonary vascular resistance. RV systolic function was evaluated through tricuspid annular plane systolic excursion (TAPSE), obtained by placing the M-mode cursor at the lateral tricuspid annulus and measuring the systolic displacement from end-diastole to end-systole. All images were acquired in accordance with the American Society of Echocardiography guidelines and stored for subsequent analysis [42].

### 2.6. Sample Collection

Twenty-four days after MCT administration, animals from all experimental groups (SC, SH, SHB, EH, and EHB) were euthanized by decapitation. This time point was selected according to previous reports [27,30,32], as it represents the average survival period of rats developing PAH after receiving a 60 mg/kg dose of MCT.

### 2.7. Lung and RV Oxidative Stress Biomarkers

#### 2.7.1. Tissue Preparation

Frozen lung and right ventricle (RV) samples (100 mg each) were homogenized in 1 mL of phosphate buffer (pH 7.4) using a Tissue Master 125 homogenizer (OMNI International, Kennesaw, GA, USA) and centrifuged at 10,000× *g* (12,000 rpm) for 10 min at 4 °C. Supernatants were collected to assess the enzymatic activities of superoxide dismutase (SOD), catalase (CAT), and glutathione S-transferase (GST), as well as the levels of malondialdehyde (MDA), nitric oxide (NO), and total protein (TP). The pellets were reserved for carbonyl protein (CP) quantification. Measurements were performed using either an ELISA microplate reader (Multiskan GO, Thermo Fisher Scientific, Waltham, MA, USA) or a UV-visible spectrophotometer (UVmini-1240, Shimadzu Corporation, Kyoto, Japan).

#### 2.7.2. Superoxide Dismutase Activity

SOD activity was quantified following the method of Dieterich et al. [43], based on the inhibition of pyrogallol autoxidation. For the assay, 30 μL of sample was combined with 99 μL of potassium phosphate buffer (5 mmol/L, pH 8.0) and 15 μL of pyrogallol (100 μmol/L). Absorbance was recorded at 570 nm. Enzymatic activity was normalized to protein content and expressed as U/mg of protein, with one unit defined as the activity required to inhibit pyrogallol autoxidation by 50%. Blank and standard preparations were analyzed in duplicate without and with pyrogallol, respectively.

#### 2.7.3. Catalase Activity

The CAT activity was measured based on an adaptation of the Hadwan method [44], using hydrogen peroxide (H_2_O_2_) as a substrate. For this, 100 μL of H_2_O_2_ (20 mmol/L) was pipetted into 5 μL of the sample. After 3 min, 150 μL of ammonium molybdate (32.4 mmol/L) was added to stop the reaction. Blank samples were prepared by replacing H_2_O_2_ with sodium and potassium phosphate buffer (50 mmol/L, pH 7.0). The reading was taken at 374 nm using a spectrophotometer. To calculate the CAT value, a standard curve was prepared with serial dilutions of H_2_O_2_. Test values were corrected by subtracting control values to eliminate interference from other serum compounds that could react with ammonium molybdate. CAT activity was expressed as CAT KU/mg of protein.

#### 2.7.4. Glutathione S-Transferase Activity

GST activity was assessed according to the method described by Habig et al. [45]. The reaction mixture contained 970 μL of 50 mmol/L buffer (pH 7.0), 10 μL of reduced glutathione (1 mmol/L), 10 μL of CDNB (1 mmol/L), and 10 μL of sample. Following the addition of CDNB, changes in absorbance were recorded at 340 nm for 60 s. Enzyme activity was calculated using a molar extinction coefficient of ε340 = 9.6 mmol/L/cm. One unit of GST activity corresponded to the amount of enzyme catalyzing the formation of 1 μmol of product per minute per milliliter, and the results were expressed as μmol/min/g.

#### 2.7.5. Nitric Oxide Production

NO production was assessed using the Griess reaction according to Tsikas [46]. Samples (50 μL) were mixed with an equal volume of Griess reagent containing 1% sulfanilamide, 0.1% N-(1-naphthyl)ethylenediamine, and 2.5% phosphoric acid, followed by incubation for 10 min at room temperature. Absorbance was then recorded at 570 nm using a microplate reader. NO levels were calculated from a sodium nitrite standard curve ranging from 0 to 100 μmol/L and expressed as μmol/L.

#### 2.7.6. Malondialdehyde Determination

Lipid peroxidation was evaluated by measuring MDA according to Buege and Aust [47]. Tissue supernatant (0.2 mL) was mixed with 0.4 mL of a solution containing 15% trichloroacetic acid, 0.375% thiobarbituric acid, and 0.6% hydrochloric acid. The reaction mixture was incubated in a water bath at 90 °C for 40 min and subsequently cooled on ice. Afterward, 0.6 mL of butanol was added, and the samples were vortexed for 2 min and centrifuged at 9000× *g* for 10 min. MDA content in the resulting supernatant was determined by absorbance at 540 nm using a microplate spectrophotometer (Multiskan GO, Thermo Fisher Scientific, Waltham, MA, USA). Concentrations were calculated from a standard curve prepared with 1,1,3,3-tetramethoxypropane and expressed as μmol/L per mg of protein.

#### 2.7.7. Carbonylated Protein Determination

Protein carbonyl (PC) content was determined using the 2,4-dinitrophenylhydrazine (DNPH) method [48]. Pellets obtained from the tissue homogenates were resuspended in 0.5 mL of 10 mmol/L DNPH prepared in 7% hydrochloric acid and incubated for 30 min at room temperature in the dark, with intermittent mixing. Subsequently, 0.5 mL of ice-cold 10% trichloroacetic acid was added, and the samples were centrifuged at 5000× *g* for 10 min at 4 °C. The resulting supernatant was discarded, and the pellet was washed three times with 1 mL of an ethyl acetate/ethanol mixture (1:1, *v*/*v*). Thereafter, 1 mL of 6% sodium dodecyl sulfate was added, and the samples were vortexed until the pellet was completely dissolved. Absorbance was measured at 370 nm, and PC levels were calculated using the molar extinction coefficient ε370 = 22 mmol/L/cm and expressed as nmol/mg of protein.

#### 2.7.8. Total Protein

Total protein determination was performed according to the method described by Lowry et al., [49], using bovine serum albumin as a standard. The obtained total protein concentrations were used to standardize the results of CAT, SOD, MDA, and PC.

### 2.8. Isolation of Right Ventricular Myocytes

RV myocytes were isolated using a protocol previously described [38]. After euthanasia, the heart was quickly dissected, dried, and weighed. The heart was then connected to a Langendorff retrograde perfusion system via the aorta, using Tyrode solution with the following components in millimoles (mM): 130 NaCl, 1.43 MgCl_2_, 5.4 KCl, 0.75 CaCl_2_, 5.0 HEPES, 10.0 glucose, 20.0 taurine, and 10.0 creatine, adjusted to pH 7.4, for about 5 min. Following this step, the solution was replaced with Tyrode containing EGTA (0.1 mM) for another 5 min. Enzymatic digestion was then performed by perfusing the heart for approximately 12 min with Tyrode solution containing type II collagenase (1 mg/mL; Worthington Biochemical Corporation, Lakewood, NJ, USA) and protease (0.1 mg/mL; Sigma-Aldrich, St. Louis, MO, USA). The RV was subsequently isolated, minced into small fragments, and transferred to a conical flask containing the same enzymatic solution. Mechanical agitation was applied for 5 min to promote cell dissociation, after which dispersed cells were separated from the remaining tissue by filtration. Following centrifugation, the isolated cells were resuspended in Tyrode solution, while the residual tissue underwent an additional cycle of mechanical dispersion. All solutions were continuously oxygenated with 100% O_2_ (White Martins Gases Industriais Ltd., Rio de Janeiro, RJ, Brazil) and maintained at 37 °C throughout the procedure. Isolated cardiomyocytes were stored at 5 °C and used within 2–3 h after isolation.

### 2.9. Measurement of Cellular Contractility

Cardiomyocyte mechanical function was evaluated by monitoring changes in cell length using an edge-detection system (IonWizard, version 6.3.4.69, IonOptix LLC, Milton, MA, USA) coupled to an inverted microscope (Nikon Eclipse TS100, Nikon Corporation, Tokyo, Japan) equipped with a 40× oil-immersion objective (S Fluor, Nikon Corporation, Tokyo, Japan), as previously described. Isolated myocytes were transferred to an experimental chamber positioned on the microscope and superfused with HEPES-buffered solution maintained at 37 °C. Cells were field-stimulated at 5 Hz using 10 V pulses with a duration of 5 ms (MyoPacer Field Stimulator, IonOptix LLC, Milton, MA, USA). Images were acquired with a MyoCam camera (IonOptix LLC, Milton, MA, USA) at a sampling frequency of 240 Hz and analyzed using IonWizard software (version 6.3.4.69, IonOptix, Milton, MA, USA). Changes in cell length during contraction and relaxation were recorded and subsequently used to determine mechanical parameters. Only viable myocytes displaying clearly defined cell borders and sarcomeric striations, remaining quiescent at rest and free of spontaneous contractions, were included in the analysis. The variables assessed were contraction amplitude, contraction velocity, and relaxation velocity.

### 2.10. Protein Expression

The expression of type 2 ryanodine receptors (RyR2), Phospholamban (PLB), Phospho-Phospholamban, sarcoplasmic/endoplasmic reticulum Ca^2+^-ATPase (SERCA2) and sodium/calcium exchanger (NCX1) proteins was determined by Western blotting, as previously described [50]. Frozen RV fragments (100 mg) were homogenized in RIPA buffer (150 mM NaCl, 50 mM Tris-HCl pH 8.0, 0.1% SDS, 1% NP-40) supplemented with protease and phosphatase inhibitors (Sigma-Aldrich, St. Louis, MO, USA; PhosSTOP, Roche Diagnostics GmbH, Mannheim, Germany). After centrifugation (3000× *g*, 4 °C, 10 min), supernatants were subjected to SDS-PAGE (8–10% gels), and proteins were transferred to nitrocellulose membranes. Equal protein loading (40 μg) was confirmed with 0.5% Ponceau S staining. Membranes were blocked (5% milk in TBS-T) for 2 h at room temperature, then incubated overnight at 4 °C with primary antibodies. Primary antibodies included α-Tubulin (#2144) and GAPDH (#97166, #97166, Cell Signaling Technology, Danvers, MA, USA), RyR2 (#TA5-87416), PLB (#PA5-85268), p-PLB (Ser16, Thr17) (#711401), SERCA2 (#MA3-919, Invitrogen, Carlsbad, CA, USA), and NCX1 (#151608, Abcam, Cambridge, UK), all at 1:1000. Secondary antibodies (IRDye 680RD or 800CW, LI-COR Biosciences, Lincoln, NE, USA) were used at 1:15,000. Bands were visualized using the Odyssey^®^ Fc Imaging System and quantified with ImageJ (version 1.8, National Institutes of Health, Bethesda, MD, USA).

### 2.11. Lung and RV Histological Analysis

Histological analyses of the lung and right ventricle (RV) followed previously established procedures [51,52,53]. Briefly, tissue fragments were fixed in Karnovsky solution (4% paraformaldehyde and 4% glutaraldehyde in 0.1 M phosphate buffer, pH 7.4) for 24 h, dehydrated in ethanol, cleared in xylene, and embedded in paraffin. Sections (5 μm) were obtained and stained with hematoxylin and eosin for the assessment of cellular and extracellular components, and with Sirius Red for collagen fiber quantification. To avoid duplicate analysis, sections were evaluated in semi-series, with one section analyzed every 10 sections.

Images were acquired using a digital camera (Olympus Q Color-3, Tokyo, Japan) coupled to a light or polarized microscope (Olympus AX-70, Tokyo, Japan) according to the staining method. Quantitative analyses were performed using ImageJ^®^ software (National Institutes of Health, Bethesda, MD, USA). In the RV, the relative proportions of cardiomyocytes (cytoplasm and nucleus), extracellular matrix, and total collagen were determined using a point-counting approach (i.e., a superimposed grid with 266 intersection points). In the lung, morphometric parameters, including alveolar septa and pulmonary alveoli, were quantified using the same approach and expressed as percentage area.

Pulmonary arteriole remodeling was additionally assessed in hematoxylin and eosin-stained sections. For each lung, nine representative fields (200× magnification) were analyzed in a blinded manner. Arteriolar wall thickness and lumen diameter were measured, and the lumen-to-wall thickness ratio was calculated as an index of vascular remodeling [36].

### 2.12. Statistical Analysis

Data were initially subjected to the Shapiro–Wilk test to assess normality. For variables with parametric distribution, a one-way analysis of variance (ANOVA) was used for group comparisons, followed by Tukey’s post hoc test when applicable. For nonparametric data, the Kruskal–Wallis test was used, followed by Dunn’s post hoc test as needed. Quantitative data are presented as mean ± standard deviation (SD), while qualitative data are expressed as percentages. A significant level of 5% (*p* < 0.05) was adopted for all analyses. Detailed information on the statistical tests applied, as well as sample sizes (i.e., n for animals and cells), is provided in the respective tables and figures. All statistical analyses were performed using GraphPad Prism^®^ software, version 10.4.0.

## 3. Results

### 3.1. PAH Establishment Indicators and Effects of Treatments

By the end of the experimental period, rats in the SH group had a lower AT/ET compared with those in the SC group (Table 1). However, the treated groups (SHB, EH, and EHB) exhibited higher values than the SH group. Likewise, TAPSE was lower in the SH, SHB, and EH groups compared with the SC group. Even so, exercised rats (EH and EHB) showed higher values than those in the SH group, and the value of the EHB group was higher than that of the SHB group. In addition, exercised animals presented a higher maximum carrying load than the non-exercised ones.

Regarding weights, rats with PAH had lower body weight compared with the control group. The heart weight of SH animals was greater compared with those of SC, SHB, EH, and EHB rats. The RV weight was higher in the hypertensive groups compared with that of the SC group. Nonetheless, treated rats (SHB, EH and EHB) displayed lower values than SH animals. The lung weight was greater in the SH and SHB groups than in the SC group, whereas the EH and EHB groups’ values were not different from that of the SC group. Similar results were observed for the lung-weight-to-tibial-length ratio. The tibial length was not different between groups. The heart-weight-to-tibial-length ratio was greater in the SH group than in the SC group, while treated groups presented lower values compared with the SH group. Similar results were observed for the RV-weight-to-tibial length ratio.

### 3.2. Lung Oxidative Stress

The activity of SOD was lower in the SH group than in the other groups (Figure 1A). In addition, CAT activity and NO levels were reduced in the SH group compared with the SC group (Figure 1B,D). The isolated treatments (SHB and EH) did not prevent these reductions, whereas the combined treatment prevented them (SH < EHB). Regarding GST activity, lower values were found in the SH, SHB, and EH groups compared with the SC group (Figure 1C), whereas the combined treatment prevented such a reduction (SH < EHB). Concerning the PC levels (Figure 1F), rats in the SH group exhibited greater values than those in the SC group; however, no treatment effects were observed. No differences between groups were found for the MDA levels (Figure 1E).

### 3.3. RV Oxidative Stress

The rats in the SH group showed decreased SOD (Figure 2A), CAT (Figure 2B), and GST (Figure 2C) activities compared with those in the SC group. However, the treatments, either alone (SHB and EH groups) or in combination (EHB group), prevented such a reduction to a similar extent. The levels of NO were also lower in the SH group than in the SC and EHB groups (Figure 2D). Concerning MDA levels (Figure 2E), they were higher in the SH group than in the SC group. As for the CP levels (Figure 2F), rats in the SH group showed increased values compared with those in the SC group, while this elevation was similarly attenuated by both interventions, either alone (SHB and EH groups) or combined (EHB group).

### 3.4. Single RV Myocyte Contractile Function

Myocytes from rats in the SH group showed a reduced contraction amplitude compared with those from the SC group (Figure 3A). However, both treatments, either alone (SHB and EH groups) or combined (EHB group), prevented such a reduction to a similar extent. The departure (Figure 3B) and return (Figure 3C) velocities of shortening were lower in myocytes from hypertensive rats (SH, SHB, EH, and EHB groups) than in those from the SC group. Nevertheless, the employed treatments either alone (SHB and EH groups) or combined (EHB groups) resulted in higher velocities compared with those observed in the SH group.

### 3.5. Expression of Ca^2+^ Handling Proteins

Concerning the expression of proteins involved in Ca^2+^ handling, the levels of RyR2 (Figure 4A) and p-PLBser16 (Figure 4C) were lower in the SH group compared with the SC, EH, and EHB groups. No differences between groups were observed in the expression of PLBt (Figure 4B) or NCX (Figure 4E). Regarding SERCA2a expression, the SH group showed reduced levels compared with those of the SC group (Figure 4D).

### 3.6. Lung Remodeling

Representative lung photomicrographs (Figure 5A) reveal maintained pulmonary architecture in the SC group, characterized by thin septa, open alveoli, and the absence of inflammatory signs. In contrast, the SH group displayed disrupted microarchitecture, with thickened alveolar septa and inflammatory infiltrates. However, these structural alterations were attenuated in all treated groups (SHB, EH, and EHB), which showed better alveolar preservation, reduced septal thickening, and less inflammation. In addition, in the lung sections in Figure 5B, it can be observed that the SH group displayed a thickened arteriolar wall compared with the SC group, while such a change was lessened in the treated groups. Moreover, lung sections in Figure 5C show augmented collagen fibers in the SH group than in the control group, and that this increase was attenuated in the treated groups. The quantitative analysis indicated that hypertensive rats (SH, SHB, EH, and EHB groups) had a higher percentage of alveolar septa (Figure 5D) and a lower percentage of pulmonary alveoli (Figure 5E) than the control rats (SC group). The diameter-to-wall thickness ratio of pulmonary arterioles was lower in rats from the SH group than in those from the SC group (Figure 5F). Nevertheless, all treatments attenuated this reduction, partially restoring the diameter-to-wall thickness ratio, although values remained lower than those observed in the SC group. Finally, total collagen was higher in rats from the SH group compared with the control ones (SC group) (Figure 5G). However, all treatments prevented such an increase.

### 3.7. RV Remodeling

Cardiac sections from the SC group (Figure 6A,B) showed preserved RV morphology, with organized fibers, central nuclei, and homogeneous cytoplasm, without inflammatory infiltration or extracellular matrix accumulation. In contrast, SH animals presented disorganized fibers, reduced cardiomyocyte density, inflammatory foci, and marked extracellular matrix and collagen deposition. These changes were attenuated in the treated groups (SHB, EH, and EHB), which displayed better conservation of tissue architecture and less fibrosis compared with the SH group, with no prominent inflammatory infiltration observed. The quantitative analysis showed that rats in the SH group had a lower percentage of cardiomyocytes (Figure 6C), and higher percentages of extracellular matrix (Figure 6D) and total collagen (Figure 6E) than those in the SC group. However, all treated groups showed increased proportions of cardiomyocytes and reduced extracellular matrix and total collagen compared with the SH group.

## 4. Discussion

We used a model of severe PAH in rats to evaluate the effects of blueberry extract and resistance exercise training on pulmonary and RV function and structure. Both interventions, either alone or in combination, mitigated cardiopulmonary oxidative imbalance and adverse structural remodeling during PAH progression. In addition, the treatments preserved RV myocyte contractile function, while resistance training, alone or combined with blueberry extract, was associated with the partial preservation of key Ca^2+^-handling proteins. Overall, these adaptations were accompanied by the attenuation of pulmonary vascular impairment and RV dysfunction.

The echocardiographic analyses confirmed that the injected dose of monocrotaline markedly increased the pulmonary artery resistance (i.e., a reduced AT/ET ratio) and reduced RV systolic function (i.e., TAPSE) in hypertensive rats, changes that are typically observed in disease progression [27,30,32,54]. Such RV dysfunction was followed by impaired RV single cardiomyocyte contractility, characterized by a reduced amplitude of shortening and slower velocities of shortening and relaxation.

In line with these functional disturbances, we observed that the induced PAH caused a pronounced oxidative imbalance (i.e., decreased SOD, CAT, GST and NO) and elevated oxidative damage (i.e., increased MDA and PC) in both the lung and RV. In addition, the lung of these rats exhibited inflammatory infiltrates and hemorrhagic alveoli, followed by alveolar septal thickening and increased collagen deposition, along with reduced alveolar density and arteriole diameter. Likewise, the RV of hypertensive rats showed structural changes like reduced proportion of myocytes, expansion of the extracellular matrix, and collagen accumulation. Moreover, the RV presented downregulation of Ca^2+^ regulatory proteins (i.e., RyR2 and SERCA2a). These changes are strongly associated with PAH progression [10,11,12,13,14,30,35]. More importantly, our results indicate that both interventions attenuated these impairments, and the observed functional benefits are believed to be associated with metabolic, mechanical and structural adaptations to the applied treatments. Initially, it is noteworthy that the resistance training protocol used here improved the rats’ exercise tolerance (i.e., maximum carrying load), although blueberry extract either alone or combined with resistance training did not affect exercise tolerance. This improvement may be attributed not only to the preservation of central parameters (i.e., lung and heart) but also to peripheral adaptations to resistance training, including the maintenance of skeletal muscle integrity and enhanced respiratory efficiency [29,55].

Regarding the cardiopulmonary functional benefits of the applied treatments (i.e., AT/ET and TAPSE), it is conceivable that the preservation of NO bioavailability provided by the treatments played a role, since resistance exercise enhances eNOS activity and antioxidants can potentiate this pathway [56,57]. Indeed, the preservation of NO levels was particularly evident with the combined intervention in both lung and RV tissues, suggesting that maintenance of NO bioavailability may contribute to the observed cardiopulmonary benefits. Moreover, the interventions attenuated the lung and RV pro-oxidant profile, with exercise showing the greatest capacity to preserve antioxidant activity. Since oxidative stress activates fibroblasts and apoptotic pathways [58,59,60], these biochemical adaptations provide a mechanistic explanation for the prevention of pathological remodeling observed in both lung and RV tissues. Really, the RV of treated rats displayed attenuated extracellular matrix and collagen accumulation and preserved architecture. Similarly, the interventions mitigated the structural changes and improved the pulmonary oxidative profile. Altogether, our data support the concept that pathological remodeling in PAH emerges as the final consequence of oxidative imbalance, and that interventions capable of restoring these upstream processes can substantially mitigate cardiopulmonary damage [30,35].

Concerning the impaired contractility of single RV myocytes and the downregulation of Ca^2+^ handling proteins, the employed treatments attenuated the reduction in cellular mechanical function, as well as the expression of RyR2 and p-PLB(Ser16), indicating the partial preservation of calcium release and reuptake. These findings align with previous studies showing that resistance training improves cardiomyocyte contractile function as well as intracellular calcium dynamics by upregulating regulatory proteins [29]. It is noteworthy that our resistance exercise protocol improved antioxidant defenses in the RV of trained rats and thus attenuated oxidative stress, which positively impacts the disruption of Ca^2+^ handling [11,58,59]. Notably, this study demonstrates for the first time that blueberry extract directly contributed to the preservation of RV myocyte function, despite having no direct impact on the expression of Ca^2+^ regulatory proteins. It is reasonable, nevertheless, that the antioxidant properties of blueberry favored RV redox regulation, which positively influenced intracellular Ca^2+^ dynamics [10,11].

Taken together, these findings indicate that blueberry extract and resistance training were associated with complementary cardiopulmonary protective responses during severe PAH progression. The combined intervention showed broader preservation of pulmonary redox markers, whereas resistance training was particularly associated with improved RV systolic function. Across the interventions, the attenuation of adverse structural remodeling and the preservation of RV myocyte contractile function support the potential relevance of these strategies in severe experimental PAH.

### Limitations

This study has some limitations that should be considered when interpreting the findings. First, in the resistance training protocol, the animals’ climbing speed could not be precisely controlled, limiting the accurate determination of exercise intensity and total workload. Second, as blueberry extract is a natural product, variations in its chemical composition associated with cultivation conditions, harvesting, and processing may influence the reproducibility and consistency of the intervention. Although the extract was stored protected from light at −80 °C and fresh working solutions were prepared daily to minimize phenolic degradation, stability and degradation kinetics were not directly assessed during the experimental period. This is particularly relevant for anthocyanins and other phenolic compounds that may undergo oxidation during processing and storage. Nevertheless, previous studies have demonstrated substantial preservation of blueberry anthocyanins and other phenolic compounds under low-temperature and light-protected storage conditions [61,62,63]. In addition, circulating concentrations of anthocyanins, phenolic acids, or their metabolites were not measured; therefore, the systemic exposure and bioavailability of the administered extract could not be directly determined. Previous evidence indicates that blueberry-derived phenolics are absorbed and metabolized in the gastrointestinal tract, with parent compounds and/or their metabolites reaching the systemic circulation [64]. Moreover, specific toxicological outcomes were not assessed in the present study; therefore, potential adverse effects associated with prolonged or high-dose exposure to concentrated polyphenol-rich extracts cannot be excluded. Although available evidence suggests limited overt toxicity following the oral intake of anthocyanin-rich extracts, the safety of high-dose polyphenol exposure remains incompletely characterized. Moreover, high-dose supplementation may exert pro-oxidant and other adverse effects depending on the dose, duration of exposure, and physiological context [65,66]. Future studies should therefore include dedicated safety and dose–response assessments, particularly when considering long-term supplementation or translation to clinical settings. Third, the duration of the interventions was constrained by the time course of severe MCT-induced PAH, which limits the extrapolation of our findings to longer-term adaptations. Finally, only male rats were included, limiting the generalizability of the findings and highlighting the need for future studies investigating sex-specific responses to these interventions in severe PAH.

## 5. Conclusions

In conclusion, blueberry extract and resistance training, either alone or in combination, alleviate cardiopulmonary oxidative imbalance and adverse structural remodeling, sustain RV myocyte contractility and the expression of proteins involved in Ca^2+^ cycling, and thus conserve pulmonary and right ventricle function in severe PAH induced by monocrotaline. These findings reinforce the relevance of integrating non-pharmacological strategies involving natural antioxidants and physical exercise in mitigating cardiopulmonary dysfunctions and structural damage associated with severe PAH, which could have significant implications for disease management and prognosis.

## Figures and Tables

**Figure 1:**
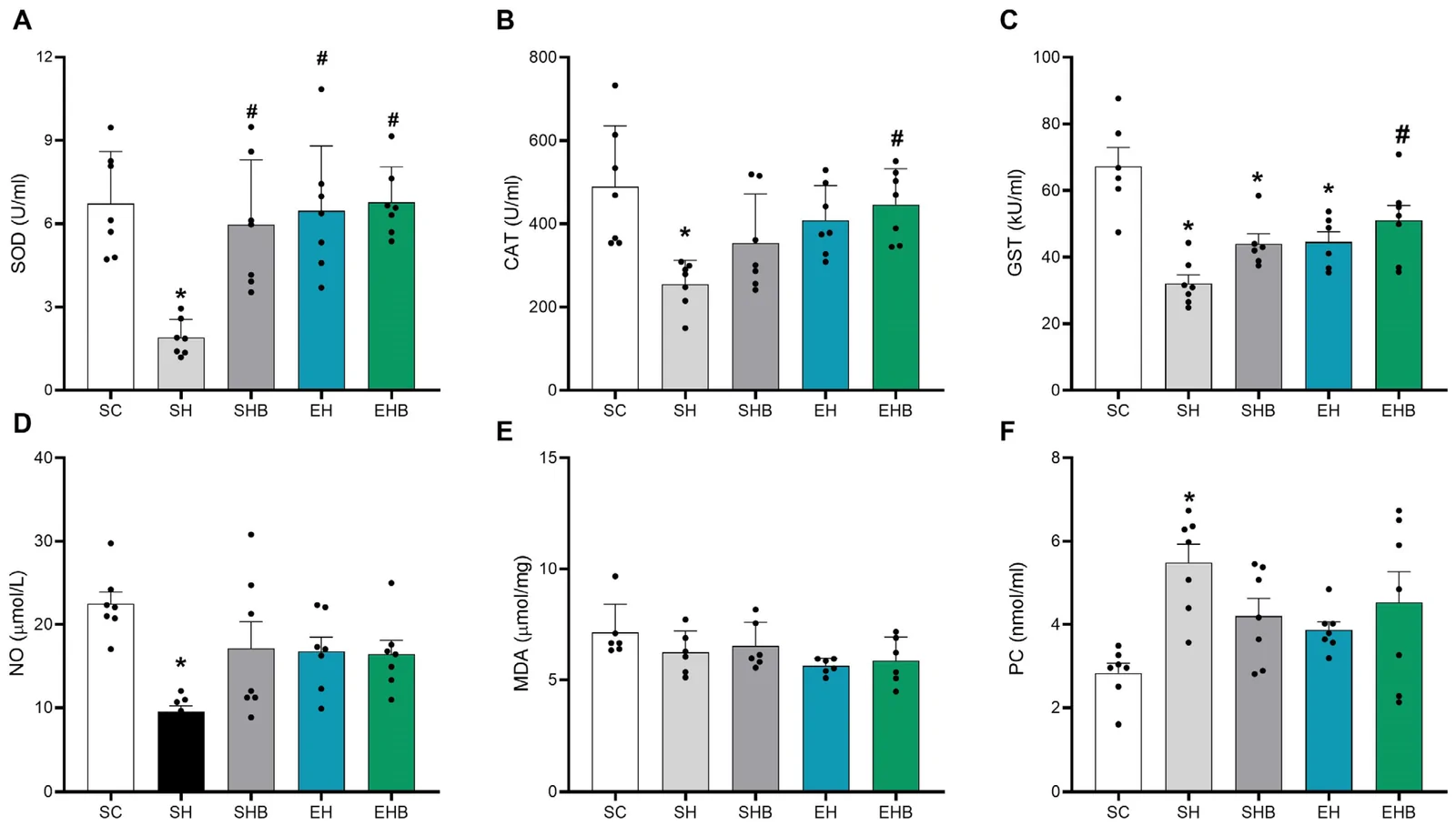
Effects of blueberry extract and resistance training on oxidative stress markers in lung tissue. (**A**) SOD (superoxide dismutase), (**B**) CAT (catalase), (**C**) GST (glutathione S-transferase), (**D**) NO (nitric oxide), (**E**) MDA (malondialdehyde), (**F**) PC (protein carbonyl). SC, sedentary control; SH, sedentary hypertensive; SHB, sedentary hypertensive blueberry; EH, exercise hypertensive; EHB, exercise hypertensive blueberry. The data are expressed as means ± SD of five to seven rats per group. One-way ANOVA followed by Tukey’s post hoc test. * *p* ˂ 0.05 vs. SC; # *p* ˂ 0.05 vs. SH.

**Figure 2:**
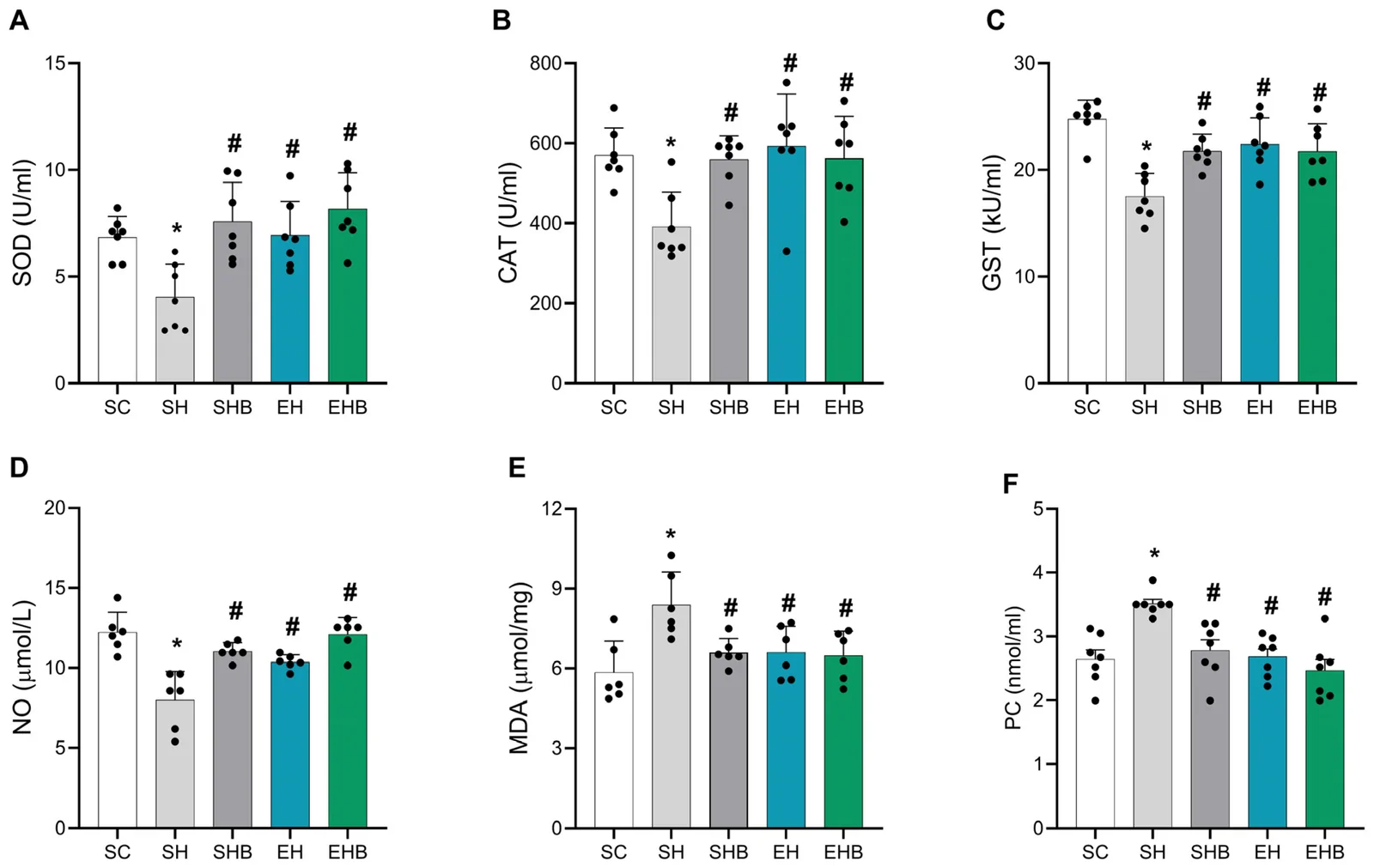
Effects of blueberry extract and resistance training on oxidative stress markers in the right ventricle. (**A**) SOD (superoxide dismutase), (**B**) CAT (catalase), (**C**) GST (glutathione S-transferase), (**D**) NO (nitric oxide), (**E**) MDA (malondialdehyde), (**F**) PC (protein carbonyl). SC, sedentary control. SH, sedentary hypertensive. SHB, sedentary hypertensive blueberry. EH, exercise hypertensive. EHB, exercise hypertensive blueberry. Data are means ± SD of five to seven rats per group. One-way ANOVA followed by Tukey’s post hoc test. * *p* ˂ 0.05 vs. SC; # *p* ˂ 0.05 vs. SH.

**Figure 3:**
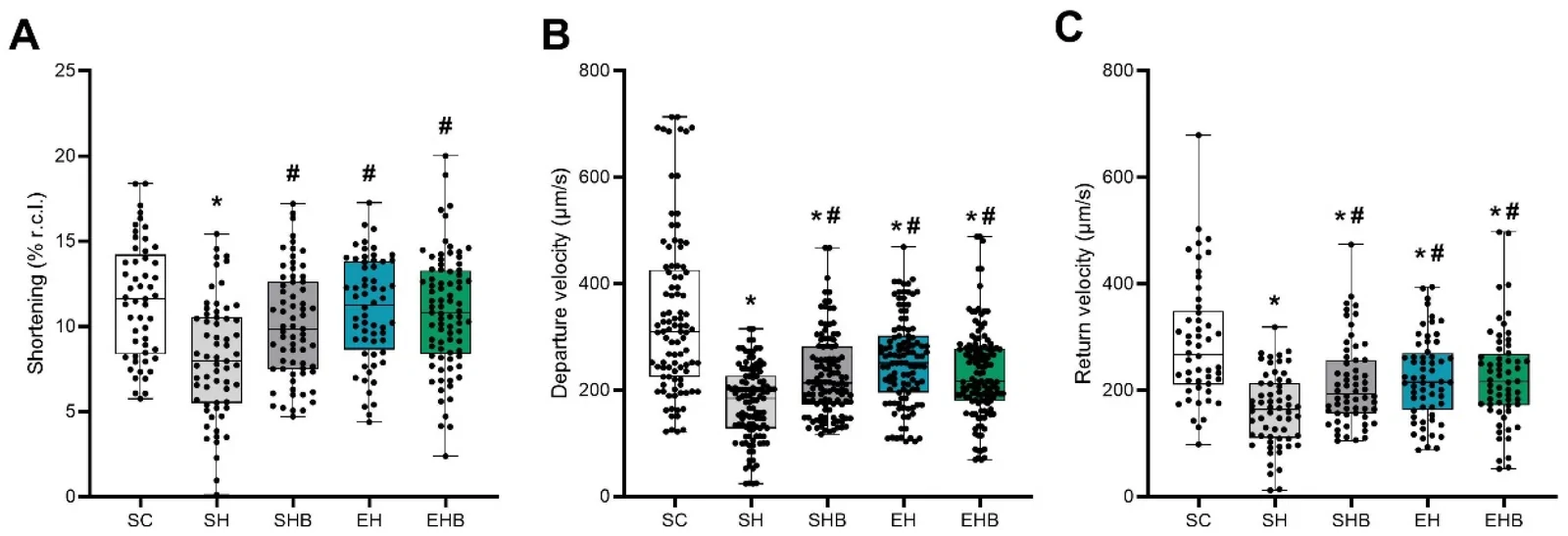
Effects of blueberry extract and resistance training on contractility in single right-ventricular myocytes stimulated at 5 Hz. (**A**) Amplitude of shortening. (**B**) Departure velocity of shortening. (**C**) Return velocity of shortening. SC, sedentary control. SH, sedentary hypertensive. SHB, sedentary hypertensive blueberry. EH, exercise hypertensive. EHB, exercise hypertensive blueberry. Data are shown as box-and-whisker plots (median, interquartile range; whiskers = minimum–maximum) overlaid with individual cell values (10 cells per animal; 5–6 animals per group). Between-group differences were assessed with the Kruskal–Wallis test followed by Dunn’s multiple comparisons. * *p* < 0.05 vs. SC; # *p* < 0.05 vs. SH.

**Figure 4:**
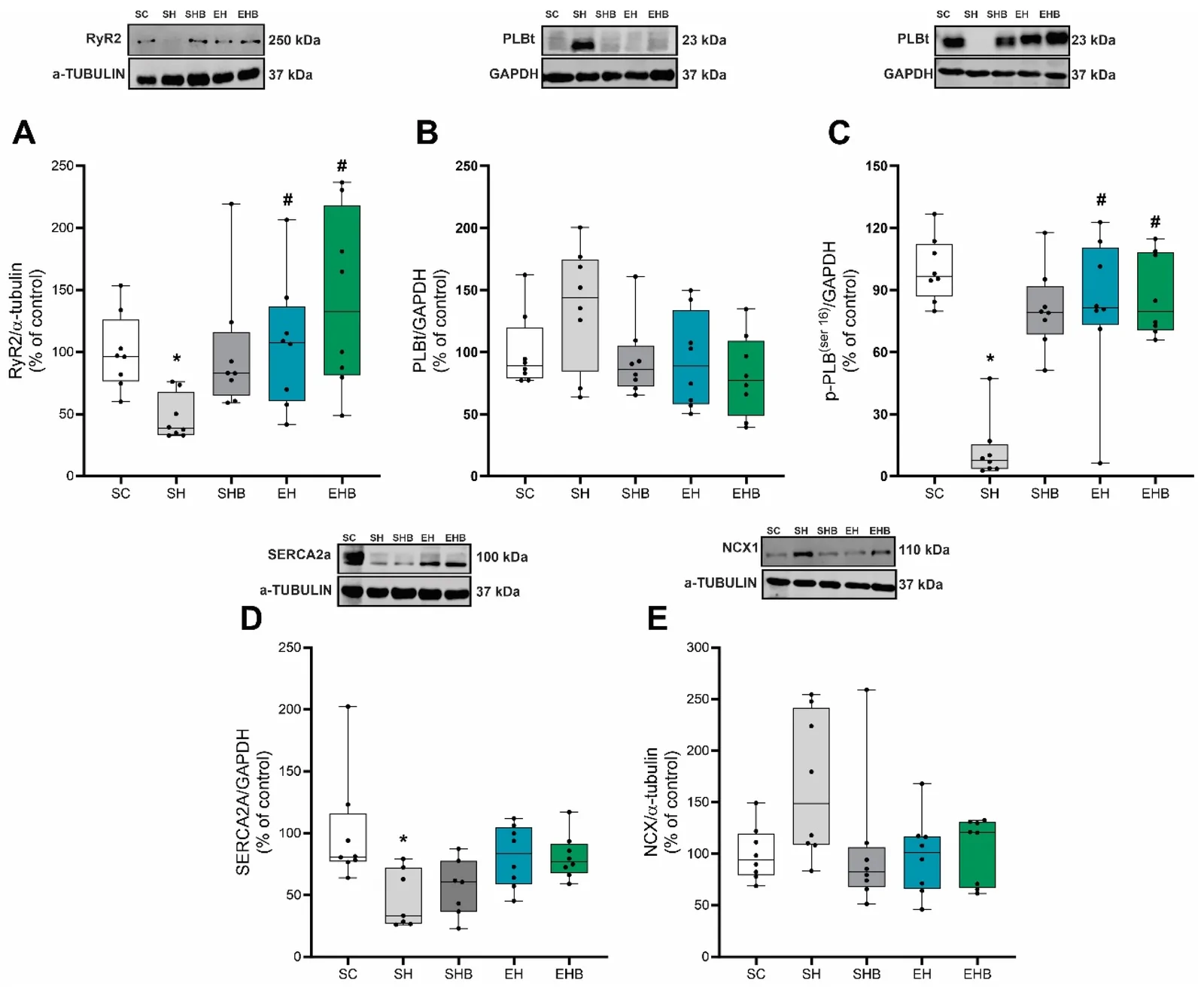
Effects of blueberry extract and resistance training on the expression of Ca^2+^-handling proteins in the right ventricle. (**A**) Ryanodine receptor type 2 (RyR2). (**B**) Total phospholamban (PLB_t). (**C**) Phospholamban phosphorylated at Ser^16^ (p-PLB^Ser16). (**D**) Sarcoplasmic reticulum calcium ATPase type 2a (SERCA2a). (**E**) Na^+^/Ca^2+^ exchanger (NCX1). SC, sedentary control. SH, sedentary hypertensive. SHB, sedentary hypertensive blueberry. EH, exercise hypertensive. EHB, exercise hypertensive blueberry. Data are shown as box-and-whisker plots (median, interquartile range; whiskers = minimum–maximum) with individual animal values (n = 7–8 per group), expressed as % of SC (control). Group differences were tested with the Kruskal–Wallis test followed by Dunn’s post hoc comparisons. * *p* < 0.05 vs. SC; # *p* < 0.05 vs. SH.

**Figure 5:**
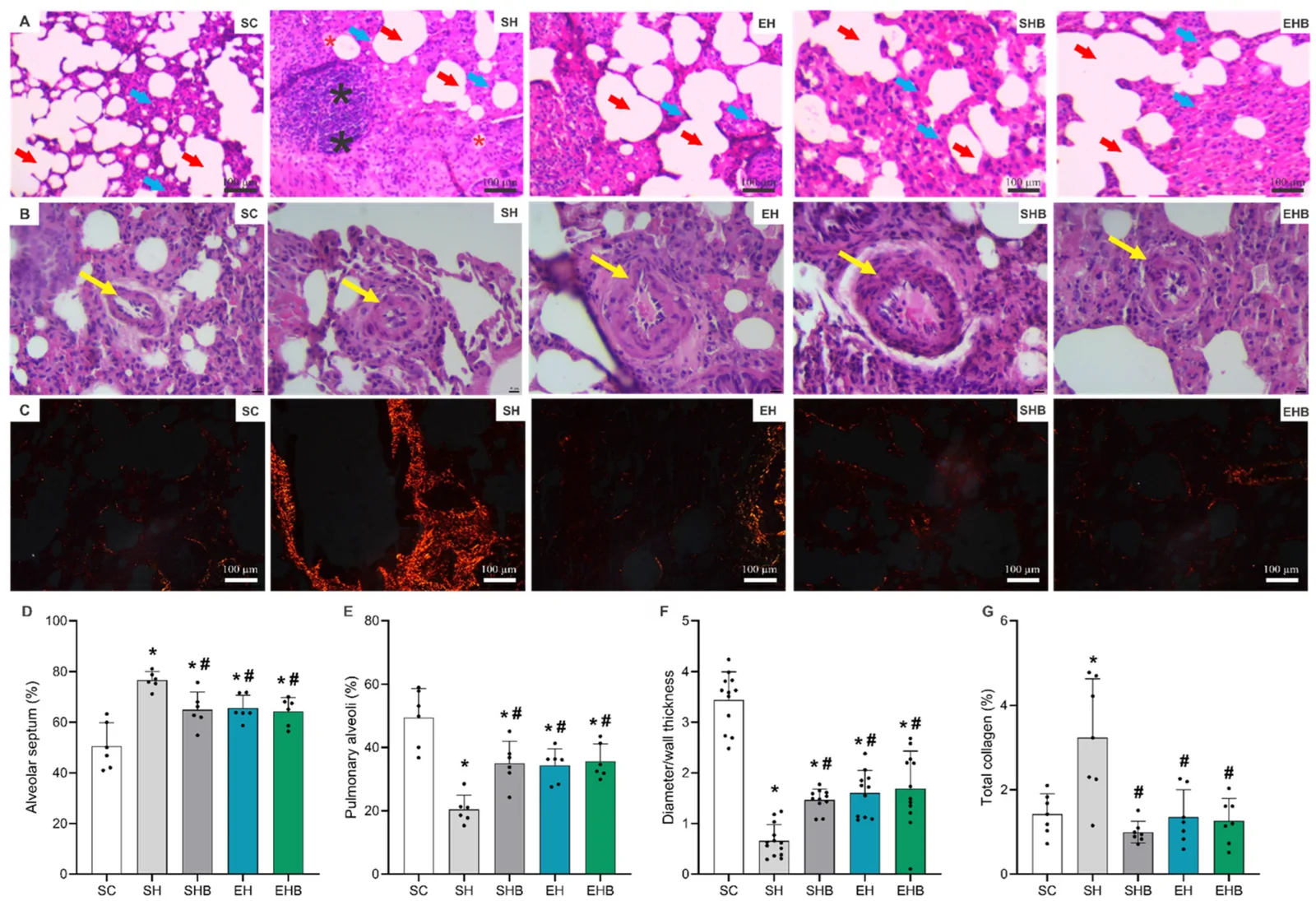
Effects of blueberry extract and resistance training on lung histomorphometry. (**A**) Representative photomicrograph of lung tissue stained with hematoxylin and eosin (scale bar: 100 μm). (**B**) Representative photomicrographs of pulmonary arterioles stained with hematoxylin and eosin (scale bar: 100 μm). (**C**) Representative photomicrographs of lung tissue stained with picrosirius red, with collagen fibers shown in red/orange (scale bar: 100 μm). (**D**) Alveolar septum (%). (**E**) Pulmonary alveoli (%). (**F**) Pulmonary arteriole diameter/wall thickness ratio. (**G**) Total collagen (%). Blue arrows: alveolar septum; red arrows: alveolus; red asterisks: hemorrhagic alveolus; black asterisks: inflammatory foci; yellow arrows: pulmonary arteriole. SC, sedentary control; SH, sedentary hypertensive; SHB, sedentary hypertensive blueberry; EH, exercise hypertensive; EHB, exercise hypertensive blueberry. Data are expressed as mean ± SD of 10 images per animal in each group (7 animals per group). One-way ANOVA followed by Tukey’s post hoc test. * *p* < 0.05 vs. SC; # *p* < 0.05 vs. SH.

**Figure 6:**
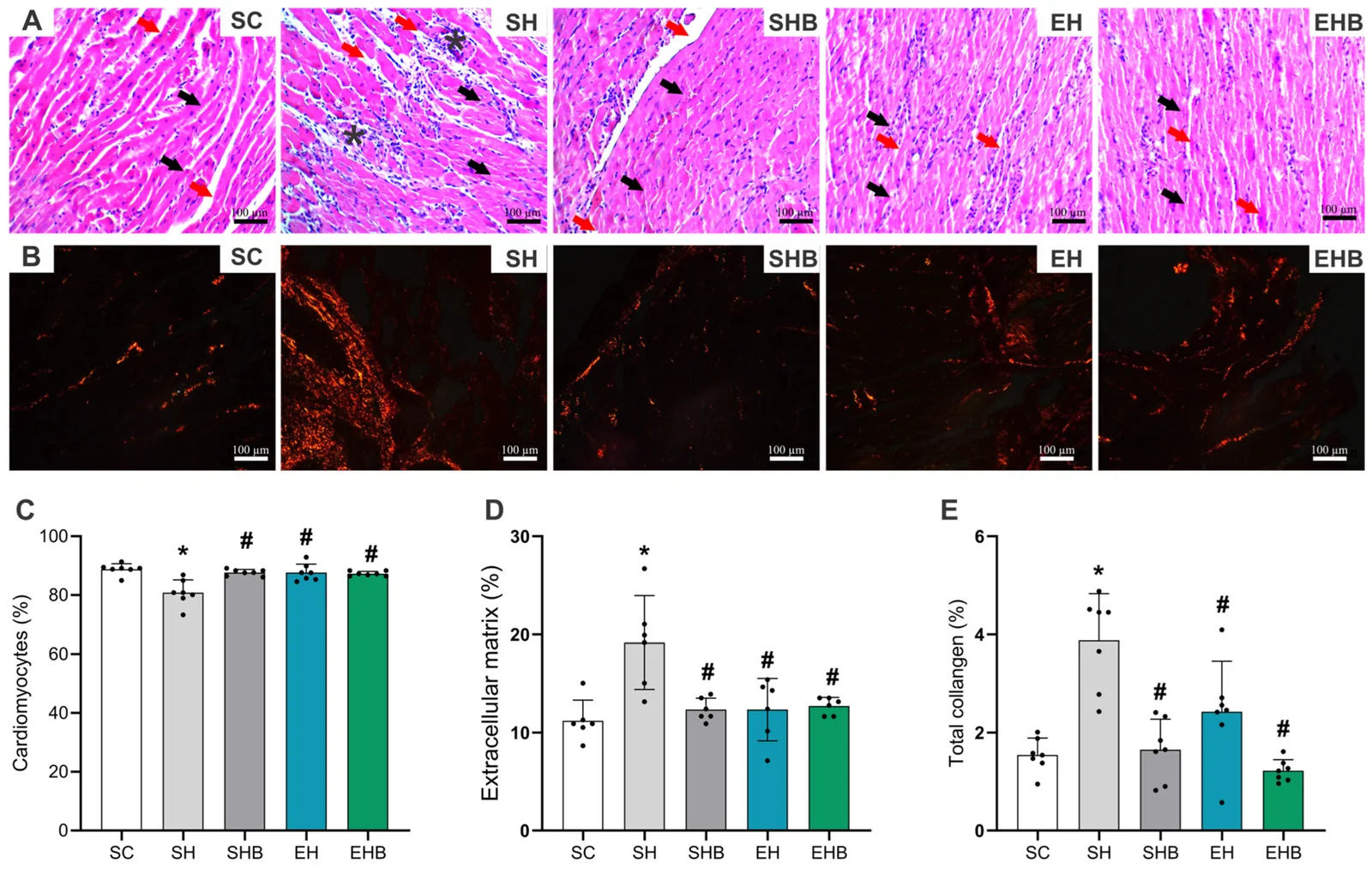
Effects of blueberry extract and resistance training on right ventricular remodeling. (**A**) Representative photomicrographs of tissue stained with hematoxylin and eosin (scale bar: 100 μm). (**B**) Representative photomicrographs of tissue stained with picrosirius red, with collagen fibers shown in red/orange (scale bar: 100 μm). (**C**) Percentage of cardiomyocytes. (**D**) Percentage of extracellular matrix. (**E**) Percentage of total collagen. Black arrows: cardiomyocytes; red arrow: extracellular matrix; asterisks: inflammatory foci. SC, sedentary control; SH, sedentary hypertensive; SHB, sedentary hypertensive blueberry; EH, hypertensive exercise; EHB, exercise hypertensive blueberry. Data are means ± SD of seven rats in each group. One-way ANOVA followed by Tukey’s post hoc test. * *p* ˂ 0.05 vs. SC; # *p* ˂ 0.05 vs. SH.

**Table 1:** Animal physical and functional parameters.

	SC	SH	SHB	EH	EHB
Initial body weight, g	201.30 ± 5.37	200.90 ± 6.64	201.10 ± 5.03	201.90 ± 3.56	200.40 ± 3.38
Final body weight, g	294.30 ± 19.92	258.50 ± 17.55 *	263.50 ± 17.14 *	259.60 ± 13.86 *	289.00 ± 15.03 *
Heart weight, g	0.97 ± 0.07	1.40 ± 0.09 *	1.04 ± 0.12 ^#^	1.01 ± 0.08 ^#^	0.99 ± 0.15 ^#^
RV weight, g	0.16 ± 0.02	0.37 ± 0.03 *	0.27 ± 0.06 *^#^	0.23 ± 0.05 *^#^	0.22 ± 0.04 *^#^
Lung weight, g	1.84 ± 0.30	3.10 ± 0.73 *	3.09 ± 0.56 *	2.68 ± 0.61	2.62 ± 0.83
Tibial length, mm	37.77 ± 0.68	36.03 ± 1.10	36.06 ± 0.87	35.77 ± 1.22	36.23 ± 1.12
Heart weight/tibial length, mg/mm	0.029 ± 0.00	0.038 ± 0.00 *	0.029 ± 0.00 ^#^	0.026 ± 0.00 ^#^	0.025 ± 0.00 ^#^
RV weight/tibial length, mg/mm	0.004 ± 0.00	0.010 ± 0.00 *	0.008 ± 0.00 *^#^	0.007 ± 0.00 *^#^	0.006 ± 0.00 *^#^
Lung weight/tibial length, mg/mm	0.05 ± 0.00	0.09 ± 0.02 *	0.09 ± 0.02 *	0.07 ± 0.01	0.07 ± 0.02
AT/ET, ms	0.60 ± 0.02	0.22 ± 0.01 *	0.39 ± 0.03 *^#^	0.45 ± 0.02 *^#^	0.44 ± 0.03 *^#^
TAPSE, mm	2.57 ± 0.12	1.55 ± 0.07 *	1.89 ± 0.08 *	2.02 ± 0.01 *^#^	2.26 ± 0.11 *^#†^
Initial maximum carrying load, g/g	1.11 ± 0.11	1.05 ± 0.08	1.01 ± 0.13	0.99 ± 0.08	0.98 ± 0.08
Final maximum carrying load, g/g	1.26 ± 0.13	1.00 ± 0.10	1.30 ± 0.19	1.74 ± 0.12 ^#^	1.74 ± 0.08 ^#^

Data are means ± SD of seven rats in each group. SC, sedentary control. SH, sedentary hypertensive. SHB, sedentary hypertensive blueberry. EH, exercise hypertensive. EHB, exercise hypertensive blueberry. AT/ET, pulmonary artery flow acceleration time (AT) to ejection time (ET) ratio. BW, body weight. Futon’s Index, right ventricle weight to interventricular septum + left ventricle weight ratio. RV, right ventricle. TAPSE, tricuspid annular plane systolic excursion. One-Way ANOVA followed by Tukey’s post hoc test. * *p* ˂ 0.05 vs. SC; ^#^ *p* ˂ 0.05 vs. SH; ^†^ *p* ˂ 0.05 vs. SHB.

## Data Availability

The data will be shared upon reasonable request to the corresponding author due to ethical restrictions related to the use of animals in research.
